# Inflammatory Serum Biomarkers in Colorectal Cancer in Kazakhstan Population

**DOI:** 10.1155/2020/9476326

**Published:** 2020-09-08

**Authors:** L. Akhmaltdinova, V. Sirota, V. Zhumaliyeva, D. Babenko, I. Kadyrova, Z. Tauesheva, D. Taizhanova, A. Ibraeva, M. Maratkyzy, A. Turmukhambetova

**Affiliations:** ^1^Shared Resource Lab of the Karaganda Medical University, Karaganda 10001, Kazakhstan; ^2^Oncology Department of the Karaganda Medical University, Karaganda 10001, Kazakhstan; ^3^Department of Internal Diseases of the Karaganda Medical University, Karaganda 10001, Kazakhstan; ^4^Science and Strategic Development Department of the Karaganda Medical University, Karaganda 10001, Kazakhstan

## Abstract

Colorectal cancer is a type of oncopathology widespread in Kazakhstan. The genetic component, as well as the possible etiopathogenetic mechanisms, is widely studied. One of the most promising areas is the study of diagnostic and prognostic possibilities of inflammatory biomarkers in patients with different degrees of tumor differentiation. The following biomarkers were included in the study panel: stem cell factor (SCF), vascular endothelial growth factor (VEGF), fibroblast growth factor (FGF2), interleukin 6 (IL6), interleukin 8 (IL8), macrophage migration inhibitory factor (MIF), soluble Fas (SFAS), soluble Fas ligand (sFASL), transforming growth factor *β* (TGF), tumor necrosis factor (TNF), TNF-related apoptosis-inducing ligand (TRAIL), and programmed death ligand 1 (PD-L1). The data of our study show that most of the basic proinflammatory cytokines are involved in the systemic process and their levels do not depend on the level of tissue differentiation. Serum PD-L1 has shown itself to be a promising marker for tumor growth, which depends on the degree of differentiation.

## 1. Introduction

Nowadays, it is recognized that inflammation plays an important role in the pathogenesis of cancer. On the one hand, chronic inflammation can trigger tumor transformation in up to 25% of cases [1]. On the other hand, inflammation induced or mediated by tumor growth affects the prognosis, outcome, effectiveness of therapy, and, of course, the histological and clinical picture [2]. Inflammation associated with tumor growth affects the innate and adaptive, cellular and humoral, local, and systemic immune responses. Factors such as host and tumor cytokines, protein inflammatory mediators, tumor cell infiltration, and systemic shift of myelocytes and lymphocytes in the blood are involved. The effects of cytokines can be opposite. Cytokines, related to T and NK lymphocytes, induce apoptosis and clearly have a protective action. Other cytokines increase vascular permeability, stimulate the synthesis of immunosuppressive and growth factors, and promote infiltration, metastasis, andprogression of tumor growth [1, 3]. Serum biomarkers cannot reflect tumor inflammation microenvironment; however, there is a cross-link between local and systemic inflammatory processes, which allows the use of blood as the least invasive way to study the characteristics of the inflammatory response. For these reasons, there is considerable potential in the topic of studying the role of inflammatory factors, their dependence on the stage, histological type, or degree of tumor differentiation.

### 1.1. Objective

The objective of this paper is to study the role of inflammatory mediators at different degrees of differentiation of colorectal cancer adenocarcinoma in patients of the Kazakh population.

## 2. Materials and Methods

In all groups of patients for all research objectives, persons of Kazakh nationality were included who identify themselves, their biological parents, and biological grandparents on the maternal and paternal side as “Kazakh Kazakh.” To conduct research, permission of the Committee on Bioethics of KSMU No. 305 dated May 19, 2017, was obtained. All patients gave informed consent to participate in the study.

The control group was represented by 97 healthy individuals matched for gender and age with the study groups. All patients of the control group underwent an express test of feces for occult blood and fibrocolonoscopy as part of the national screening; thus, the control group did not include patients with colorectal cancer, polyposis, or other defects of the colon or rectum.

The experimental group consisted of 216 patients diagnosed with colorectal cancer, excluding the hereditary form of CRC. All patients underwent a general clinical examination at the outpatient stage. Morphological verification of tumors was carried out in the cytological and morphological laboratories of the Regional Clinical Oncology Center, Karaganda. The clinical diagnosis was established according to ICD 10; the TNM classification developed by the International Cancer Union was used to classify the stage of cancer and the degree of differentiation. The study included patients with adenocarcinoma, which are divided into groups according to the degree of histological differentiation: G1 (highly differentiated), G2 (moderately differentiated), and G3 (low differentiated). For age median (Me), 1st and 3d quartiles are presented. The characteristics of the experimental group are demonstrated in Table 1.

Blood sampling was standardly carried out in the morning, on an empty stomach, before surgery.

For the study, we used the Milliplex Map (Millipor) Human Circulation Biomarker panel in blood serum by an immunofluorescence method using XMap technology, which included simultaneous immunofluorescence determination of the following analytes on magnetic spheres: AFP, VEGF-A, FGF-2, IL-6, IL-8, MIF, sFas, sFasL, TGF, TNF, TRAIL, and PD-L1.

Statistical analysis was carried out in the programs R statistics (Compare Groups R packages) and SPSS 26 (IBM). The nonparametric Kruskal–Wallis criteria were used; for intergroup comparison, the Mann–Whitney *U* test with the Holm correction for multiple comparisons was used.

## 3. Results

The results showed that inflammatory markers and apoptosis markers generally respond to the tumor process; in some cases, the degree of tumor differentiation affects the profile of mediators. The results are presented in Table 2.

Table 2 demonstrates median (Me), 1st and 3rd quartiles of biomarkers in groups, and *p* value for Kruskal–Wallis comparison criteria. Intergroup comparison of the Mann–Whitney *U* test *p* value with the Holm correction presented in the text does not complicate the table.

Stem cell factor (SCF), vascular endothelial growth factor (VEGF), fibroblast growth factor (FGF2), interleukin 6 (IL6), interleukin 8 (IL8), macrophage migration inhibitory factor (MIF), soluble Fas (SFAS), soluble Fas ligand (sFASL), tumor growth factor (TGF), tumor necrosis factor (TNF), TNF-related apoptosis-inducing ligand (TRAIL), and programmed death ligand 1 (PDL1).

The system level of SCF showed no differences. The level of VEGF has a close relationship with the development of a tumor and its growth, but it is more about the local expression; the systemic level did not change in any of the groups in our study. FGF-2 increases in groups with colorectal cancer and reaches significance in the group with moderately differentiated adenocarcinoma (G1 versus control *p* = 0.9; G2 versus control *p* = 0.014; G3 versus control *p* = 0.20).

IL-6 showed the most striking differences with the control group (G1 versus control *p* = 0.018; G2 versus control *p* < 0.001; G3 versus control *p* = 0.018), but there were no differences between different degrees of differentiation. At the same time, the IL-8 did not react in any case, such as TGF and TRAIL. And TNF to a greater extent begins to react with moderate- and low-differentiated tumor tissue (G1 versus control *p* = 0.1; G2 versus control *p* < 0.001; G3 versus control *p* = 0.004). MIF showed the most interesting results. Its serum level decreases in all groups (G1 versus control *p* = 0.03; G2 versus control *p* = 0.0006; G3 versus control *p* = 0.018), but no statistical differences between the levels were obtained. Serum levels of apoptosis markers sFas/FasL showed no differences between the groups. While PDL1 shows rapid growth, the size of the groups does not allow reaching significance with G1; however, while G2 versus control *p* = 0.0044, G3 versus control *p* = 0.0032.

## 4. Discussion

The influence of the inflammatory reaction in the pathogenesis of cancer is no longer negated, but there is still insufficient knowledge to direct this process in the right direction and use it for diagnosis, prognosis, and the development of effective therapeutic strategies. Our study aimed to find out whether the degree of differentiation of pathological tissue affects the ability to cause systemic changes in inflammatory status.

Stem cell factor (SCF) is an important growth factor for hematopoietic progenitor cells with proliferative and antiapoptotic functions [4]. Growth factors, including stem cell factor, regulate the migration of tumor cells in almost all types of cancer [5]. It would be expected to see that a low degree of tumor differentiation produces more stem cell factor, but this was not recorded in our study.

VEGF as a factor of angiogenesis plays a significant role in tumor growth, which is successfully used in targeted therapy, including with CRC. Some authors find close relationships between serum and tumor levels of VEGF [6]. Serum portal vein VEGF levels in patients with CRC can be used as effective indicators for assessing CRC prognosis and predicting metastases in CRC [7]; however, not all authors find the prognostic/diagnostic role of this marker in CRC [8]. And in our study, the systemic level of VEGF does not change in groups of cancer patients.

Fibroblast growth factor (FGF) regulates adhesion, migration, and invasion in the lymph nodes [9]. FGF2 is necessary to maintain self-renewal in adult and embryonic stem cells, including cancer stem cells (cancer stem cells) [10]. In our study, FGF-2 showed differences in the group of patients with moderately differentiated carcinoma, but not in other groups.

Excessive expression of IL-6 is the most common finding when investigating the association of inflammation and cancer. IL-6 regulates numerous signaling pathways, including apoptosis, survival, proliferation, angiogenesis, invasion, and metastasis [11–13]. In a number of studies, IL-6 acts as a protector of cancer cells from therapy-induced DNA damage, oxidative stress, and apoptosis, contributing to repair [11]. In our study, it was IL-6 that showed a significant increase in each group with colorectal cancer, without any connection with the degree of differentiation.

Interleukin 8 (IL-8) similarly to IL-6 is involved in the progression of tumor growth and promotes angiogenesis, proliferation, and migration of cancer cells. It was shown its diagnostic role in the combination panel [14] and the prognostic role in the development of resistance to therapy [15]. But in our study, its serum level does not change in groups of patients compared with a group of healthy individuals.

Another important player in the inflammatory team is TNF-alpha that also plays a dual role in carcinogenesis, while being both a procarcinogen and an anticarcinogen [16]; it has been discovered by a number of researchers both in blood serum [14] and its expression in the tumor [17]. Our data show that TNF is more elevated in patients with moderate- and low-differentiated adenocarcinoma.

The immune cytokine tumor necrosis factor associated with apoptosis-inducing ligand (TRAIL) has received considerable attention as a therapeutic agent for cancer due to its ability to selectively induce apoptosis of cancer cells without causing toxicity in vivo [18, 19]. The ability of TRAIL to induce the death of cancer cells without cellular toxicity makes TRAIL a promising therapeutic agent for a wide range of cancers [18]. In our studies, we did not find a significant change in serum levels of either TRAIL or TGF-*β* in any of the groups.

MIF pleiotropic cytokine is considered key in the initiation of the inflammatory response and stimulates the production of other proinflammatory cytokines. Numerous studies show that high MIF levels are found in almost all types of human cancers and are likely to be involved in all stages of tumor development. MIF production is triggered by an autocrine signal emitted by tumor cells and stimulates the production of cytokines, chemokines, and growth, as well as angiogenic factors that lead to tumor growth, increasing its aggressiveness and metastatic potential [20, 21]. But in our study, in the examined patients in serum, we found a decrease in the level in all groups of patients. But there are no significant differences between groups with different levels of differentiation.

Fas signaling promotes metastasis of colorectal cancer (CRC) by inducing an epithelial-mesenchymal transition (EMT) [22]. Fas/FasL-mediated mechanism of tumor cell destruction is involved in cancer therapy mechanisms [23], as well as in the course of an immune response against tumor tissue [24]. Our data indicate that serum levels of sFas/FasL apoptosis markers did not show differences between groups.

The PD-1/PDL-1 system is one of the most notable breakthroughs in understanding the nature of cancer in recent years [25]. A flurry of research followed the 2018 Nobel Prize. Expression of programmed death ligand 1 (PD-L1) is often observed in human cancer. The binding of PD-L1 to its PD-1 receptor on activated T cells inhibits antitumor immunity by counteracting T-cell activating signals [26]. In our study, we found a significant increase in serum ligand level in all groups, there is a tendency to increase the level with a decrease in the degree of differentiation, but this did not become statistically significant.

## 5. Conclusion

All types of tumors are an inducer of the inflammatory response, but at the systemic level, we see only the most key events in the pathogenesis of tumor growth; the data of our study show that the most basic proinflammatory cytokines are involved in the systemic process, and in most cases their level does not depend on the level differentiation of tissue. But the tumor necrosis factor mostly increases only in moderately and low differentiated adenocarcinomas. It is difficult for us to explain a decrease in the MIF level, which contradicts with generally accepted data; perhaps this is a good sign, an adaptive decrease that the immune system is trying to take to reduce the role of procarcinogenic proinflammatory cytokines. Serum PD-L1 has shown itself to be a promising marker for tumor growth, which may depend on the degree of differentiation. In general, the small dependence of the systemic inflammatory response on the degree of differentiation in the clinical sense is a good sign; this can convince us that the level of differentiation does not affect the diagnostic or prognostic role of markers, or the strategy for the development of therapy.

## Figures and Tables

**Table 1:** Characteristics of patients in the experimental group.

Degrees of differentiation	G1	G2	G3	Control group
*n*	16	175	25	97
Male	37.5%	54.2%	44%	35%
Female	62.5%	45.7%	56%	65%
Age (Me[Q1; Q3])	65 [58, 68, 5]	67 [60, 5; 73]	65 [59; 68]	58 [45; 67]
Stages				
I	81.3%	13.1%	12.0%	—
II	18.7%	68%	48.0%	
III		13.7%	28%	
IV		4.0%	12.0%	

In square brackets, 1st and 3rd quartile are presented.

**Table 2:** The content of biomarkers in the studied groups.

Serum biomarkers (pg/mL)	Groups (Me[Q1; Q3])				KW *p* value
	Control	G1	G2	G3	
SCF	52, 2 [38, 250, 65, 11]	54, 17 [34, 3, 67, 2]	52, 5 [34, 9, 64, 05]	50, 75 [28, 75; 66, 93]	0.77
VEGF	89, 5 [55, 68; 121, 56]	94, 47 [60, 17, 140, 5]	108, 6 [80, 79; 158, 40]	98, 62; [90, 34; 127, 48]	0.07
FGF2	89, 5 [64, 5, 190, 6]	111, 68 [91, 15; 749, 70]	**136, 5** [**78, 0, 273, 0**]^*∗*^	128, 0 [69, 2; 813, 7]	0.024
IL6	2, 13 [0, 00; 7, 39]	**6, 8 [6, 6; 12, 30] ** ^*∗*^	**9, 05** [**2, 9, 13, 06**]^*∗*^	**7, 73** [**5, 9, 10, 5**]^*∗*^	0.001
IL8	14, 24 [8, 87, 39, 10]	12, 07 [7, 89; 46, 41]	19, 90 [10, 78; 41, 46]	20, 49 [5, 4, 61, 04]	0.55
MIF	401, 7 [211, 2, 728, 0]	**116, 7** [**83, 8, 317, 9**]^*∗*^	**215, 8** [**131, 8, 424, 3**]^*∗*^	**195, 3** [**111, 5, 361, 2**]^*∗*^	0.001
SFAS	1870, 5 [1355, 7, 2427, 4]	1672, 4 [1453, 9, 3165, 4]	2037, 1 [1547, 2, 2857, 2]	1483, 3 [1394, 1, 1696, 4]	0.015
sFASL	42, 14 [25, 4; 59, 78]	46, 58 [16, 1; 67, 51]	54, 54 [29, 29, 76, 16]	54, 54 [39, 39; 61, 81]	0.23
TGF	9, 8 [5, 0, 15, 01]	6, 23 [2, 62, 14, 02]	9, 7 [4, 32; 14, 70]	8, 15 [5, 21; 11, 92]	0.42
TNF	6, 10 [4, 31; 8, 71]	7, 79 [5, 52; 8, 89]	**8, 5 [5, 73; 12, 35] ** ^*∗*^	**8, 3 [6, 86; 10, 35] ** ^*∗*^	0.0028
TRAIL	108, 94 [73, 45; 143, 73]	113, 75 [50, 25;157, 99]	102, 6 [65, 75; 139, 62]	79, 72 [50, 37; 109, 28]	0.13
PDL1	10, 25 [7, 12, 40, 4]	30, 36 [1, 4; 38, 46]	**33, 14** [**3, 5, 44, 0**]^*∗*^	**35, 42 [20, 9; 46, 39] ** ^*∗*^	0.003

Notes: KW: Kruskal–Wallis test *p* value. Values in bold indicate significant difference at ^*∗*^*p* < 0.05.

## Data Availability

Data are available upon request to the corresponding author.

## References

[1] Murata M. (2018). Inflammation and cancer. *Environmental Health and Preventive Medicine*.

[2] Diakos C. I., Charles K. A., Mcmillan D. C., Clarke S. J. (2014). Cancer-related inflammation and treatment effectiveness. *The Lancet Oncology*.

[3] Francescone R., Hou V., Grivennikov S. I. (2014). Microbiome, inflammation, and cancer. *The Cancer Journal*.

[4] Smith M. A., Court E. L., Smith J. G. (2001). Stem cell factor: laboratory and clinical aspects. *Blood Reviews*.

[5] Vazquez-Mellado M. J., Monjaras-Embriz V., au L. (2017). Erythropoietin, stem cell factor, and cancer cell migration. *Vitamins and Hormones*.

[6] Goulart A., Ferreira C., Rodrigues A., Coimbra B., Sousa N., Leão P. (2019). The correlation between serum vascular endothelial growth factor (VEGF) and tumor VEGF receptor 3 in colorectal cancer. *Annals of Surgical Treatment and Research*.

[7] Xu H., Sun X., Sun W. (2018). Expression and clinical correlation of NGAL and VEGF in endometrial carcinoma. *European Review for Medical and Pharmacological Sciences*.

[8] Karpuz T., Araz M., Korkmaz L. (2019). The prognostic value of serum Semaphorin3A and VEGF levels in patients with metastatic colorectal cancer. *Journal of Gastrointestinal Cancer*.

[9] Jibiki N., Saito N., Kameoka S., Kobayashi M. (2014). Clinical significance of fibroblast growth factor (FGF) expression in colorectal cancer. *International Surgery*.

[10] Otte J., Dizdar L., Behrens B. (2019). FGF signalling in the self-renewal of colon cancer organoids. *Scientific Reports*.

[11] Kumari N., Dwarakanath B. S., Das A., Bhatt A. N. (2016). Role of interleukin-6 in cancer progression and therapeutic resistance. *Tumor Biology*.

[12] Kampan N. C., Xiang S. D., Mcnally O. M., Stephens A. N., Quinn M. A., Plebanski M. (2018). Immunotherapeutic interleukin-6 or interleukin-6 receptor blockade in cancer: challenges and opportunities. *Current Medicinal Chemistry*.

[13] Liu X., Qiao T., Chen W. (2020). Identification of crucial genes and pathways associated with colorectal cancer by bioinformatics analysis. *Oncology Letters*.

[14] Yamaguchi M., Okamura S., Yamaji T. (2019). Plasma cytokine levels and the presence of colorectal cancer. *PLoS One*.

[15] Burz C., Bojan A., Balacescu L. (2019). Interleukin 8 as predictive factor for response to chemotherapy in colorectal cancer patients. *Acta Clinica Belgica*.

[16] Montfort A., Colacios C., Levade T., Andrieu-Abadie N., Meyer N., Segui B. (2019). The TNF paradox in cancer progression and immunotherapy. *Frontiers in Immunology*.

[17] Obeed O. A. A., Alkhayal K., Al Sheikh A. (2014). Increased expression of tumor necrosis factor-*α* is associated with advanced colorectal cancer stages. *World Journal of Gastroenterology*.

[18] Guimarães P. P. G., Gaglione S., Sewastianik T., Carrasco R. D., Langer R., Mitchell M. J. (2018). Nanoparticles for immune cytokine TRAIL-based cancer therapy. *ACS Nano*.

[19] Nobre C. C. G., de Araújo J. M. G., Fernandes T. A. A. d. M. (2017). Macrophage migration inhibitory factor (MIF): biological activities and relation with cancer. *Pathology & Oncology Research*.

[20] O’reilly C., Doroudian M., Mawhinney L., Donnelly S. C. (2016). Targeting MIF in cancer: therapeutic strategies, current developments, and future opportunities. *Medicinal Research Reviews*.

[21] Chen J., Wang Y., Zhuo L. (2017). Fas signaling induces stemness properties in colorectal cancer by regulation of Bmi1. *Molecular Carcinogenesis*.

[22] Cacan E., Ozmen Z. C. (2020). Regulation of Fas in response to bortezomib and epirubicin in colorectal cancer cells. *Journal of Chemotherapy*.

[23] Yajima T, Hoshino K, Muranushi R (2019). Fas/FasL signaling is critical for the survival of exhausted antigen-specific CD8^+^ T cells during tumor immune response. *Molecular Immunology*.

[24] Thomas D. S., Fourkala E.-O., Apostolidou S. (2015). Evaluation of serum CEA, CYFRA21-1 and CA125 for the early detection of colorectal cancer using longitudinal preclinical samples. *British Journal of Cancer*.

[25] Gao Y., Wang J., Zhou Y., Sheng S., Qian S., Huo X. (2018). Evaluation of serum CEA, CA19-9, CA72-4, CA125 and ferritin as diagnostic markers and factors of clinical parameters for colorectal cancer. *Scientific Reports*.

[26] Sun C., Mezzadra R., Schumacher T. N. (2018). Regulation and function of the PD-L1 checkpoint. *Immunity*.

